# *Xenopus* Oocytes: A Tool to Decipher Molecular Specificity of Insecticides towards Mammalian and Insect GABA—A Receptors

**DOI:** 10.3390/membranes12050440

**Published:** 2022-04-19

**Authors:** Anaïs Bertaud, Thierry Cens, Rosanna Mary, Matthieu Rousset, Elodie Arel, Jean-Baptiste Thibaud, Michel Vignes, Claudine Ménard, Sébastien Dutertre, Claude Collet, Pierre Charnet

**Affiliations:** 1 IBMM, UMR 5247 CNRS, Université de Montpellier, ENSCM, 1919 Route de Mende, 34293 Montpellier, France; anais.bertaud@umontpellier.fr (A.B.); thierry.cens@inserm.fr (T.C.); rosana.mary@umontpellier.fr (R.M.); matthieu.rousset@inserm.fr (M.R.); elodie.arel@igf.cnrs.fr (E.A.); jean-baptiste.thibaud@supargo.fr (J.-B.T.); michel.vignes@umontpellier.fr (M.V.); claudine.menard@umontpellier.fr (C.M.); sebastien.dutertre@umontpellier.fr (S.D.); 2 INRAE, UR 406, Abeilles et Environnement, Domaine St. Paul, Site Agroparc, 84140 Avignon, France; claude.collet@inrae.fr

**Keywords:** heterologous expression, human, pharmacology, phytopharmaceuticals

## Abstract

The number of insect GABA receptors (GABAr) available for expression studies has been recently increased by the cloning of the *Acyrthosiphon pisum* (pea aphid) RDL subunits. This large number of cloned RDL subunits from pest and beneficial insects opens the door to parallel pharmacological studies on the sensitivity of these different insect GABAr to various agonists or antagonists. The resulting analysis of the molecular basis of the species-specific GABAr responses to insecticides is necessary not only to depict and understand species toxicity, but also to help at the early identification of unacceptable toxicity of insecticides toward beneficial insects such as *Apis mellifera* (honeybees). Using heterologous expression in *Xenopus laevis* oocytes, and two-electrode voltage-clamp recording to assess the properties of the GABAr, we performed a comparative analysis of the pharmacological sensitivity of RDL subunits from *A. pisum*, *A. mellifera* and *Varroa destructor* GABAr to three pesticides (fipronil, picrotoxin and dieldrin). These data were compared to similar characterizations performed on two *Homo sapiens* GABA-A receptors (α_2_β_2_γ_2_ and α_2_β_2_γ_2_). Our results underline a global conservation of the pharmacological profiles of these receptors, with some interesting species specificities, nonetheless, and suggest that this approach can be useful for the early identification of poorly specific molecules.

## 1. Introduction

In insects, γ-aminobutyric acid (GABA) binding to GABA receptors (GABAr) is the main inhibitory pathway in neuronal signaling and is central in all neuronal functions. As such, GABAr has been a prime target for several classes of insecticides or toxins [1,2]. 

The GABAr are members of the vast Cys-loop ligand gated-ion channels (CysLGIC) family [3,4]. They are homo- or hetero-pentamers of transmembrane subunits arranged around a central pore, selective for chloride, and open upon GABA binding. The main RDL (that stands for “resistant to Dieldrin”) subunit can form homopentamers and recent studies allowed the identification and cloning of several RDL genes, one in *Apis mellifera or Drosophila melanogaster (D. melanogaster)* [5,6], four in *Varroa destructor* [7] and two in *Acyrthosyphon pisum* [8], for example. Up to three other GABAr subunits can also be found in some species (named GRD–GABA-glycine Receptor-like subunit of *Drosophila*-; LCCH3–Ligand-gated Chloride Channel Homologue 3- and CG8916) that can form heteromers with RDL [9,10], although this does not constitute an absolute rule and the *A. pisum* genome, for example, does contain two genes encoding for the RDL subunit without any other GABAr subunit [8]. The formation of these heteromers, however, has not been identified in vivo so far. 

GABAr-forming RDL subunits have been mainly used in almost all the studies looking at the pharmacological sensitivity of the GABA response in insects [1,11,12]. All these RDL subunits display the same structural arrangement. They have four transmembrane segments (TM1-4), with a large extracellular N-terminal sequence beholding a conserved di-Cys-loop between 2 cysteines separated by 13 amino acids, which constitute the signature of the cys-LGIC family [1,3]. In *A. pisum*, the two genes cloned encode for GABAr RDL1 and RDL2 subunits [8] and produce, by via alternative splicing and sequence variations, at least four different variants among which three were partially characterized [8].

The growing number of RDL genes characterized from different insect species, which can be considered as a model for native GABAr in insects [1], offers the opportunity to compare their biophysical, and most importantly their pharmacological, properties. This in vitro analysis of the potential insecticide selectivity toward the RDL subunits of these different species should bring some clues to understand any differential toxicity toward these insect species in vivo. The *Xenopus laevis* oocyte expression system, which combines easy expression of exogenous GABAr on cells devoid of such endogenous receptors and the possibility to apply a large number of electrophysiological techniques, is clearly the system of choice for such pharmacological studies on insect receptors. In fact, most studies on insect receptors uses this system (see, for example, [8,13,14,15] for GABAr). As a step toward this goal, we have recently cloned the *V. destructor* RDL1, RDL2, RDL3, and RDL4 as well as the *A. mellifera* RDL subunits [7,10]. In the present work, we cloned the *A. pisum* RDL1 GABAr subunit, performed biophysical characterization, at the whole-cell and single-channel level, and produced a comparative pharmacological analysis of its sensitivities to GABA, fipronil, picrotoxin and dieldrin with those of the varroa mite and honeybee receptors. The properties of two human GABA-A receptors (α_2_β_2_γ_2_ or α_4_β_2_γ_2_ receptors) were also analyzed. These pesticides have been shown to be highly toxic for insects [16] and humans [17] and have been withdrawn from the market for most usage, but are still present in veterinary pharmacy (fipronil) or in human therapy (picrotoxin) against barbituric intoxication, for example. Precisely knowing the effects of the GABAr of species that may be in contact with them is, therefore, a key point for understanding their differential toxicity and possibly identifying molecular differences to explain it.

At a time when the European Food Safety Authorities are looking for alternative methods to evaluate phytopharmaceutical products for crop protection, this type of comparative pharmacology may be pertinent to understand the global toxicity of these products.

## 2. Materials and Methods

### 2.1. Cloning of the Ap-RDL1 and Hs-a_4_ Receptors

Total RNA from *A. pisum* was a gift from Professor. A.M.R Gatehouse (University of NewCastle). First-strand cDNA was obtained by reverse transcription of total RNA using Oligo-dT primers and Superscript II Reverse Transcriptase (Life Technologies) according to the manufacturer’s instructions, as previously described [18]. Oligonucleotides were designed to PCR amplify the whole coding sequence of *Ap*-RDL1 based on a sequence identified by Dale et al. [19] and deposited in GenBank under the accession number XM_001947090.3. Human cDNA coding for GABAR-α_4_ subunit was obtained from the hORFeome database (accession number DQ891679). The cDNA coding for *Ap*-RDL and *Hs*-α_4_ were subcloned in the pcDNA3.1(+) vector, with the Alfalfa Mosaic Virus (AMV) sequence immediately before the start codon and the 3′-UTR sequence of the *X. leavis* beta-globin gene immediately after the stop codon. *H. sapiens Hs*-α_2_, *Hs*-γ_2_ and *Hs*-β_2_, *A. mellifera Am*-RDL and *V. destructor Vd*-RDL1, *Vd*-RDL2 and *Vd*-RDL3 GABAr cDNAs were obtained in previous works [7,10,20]. Sequence alignments were completed using Vector NTI (Invitrogen, Carlsbad, CA, USA), and potential signal peptides were searched with the Interproscan prediction software (European Bioinformatics Institute in Cambridge, UK).

### 2.2. RNA Preparation and Xenopus Oocyte Isolation and Injection

cRNAs were in vitro synthesized from linearized plasmids using the M-message M-machine transcription kit (Thermo Fisher, Waltham, MA USA) following the manufacturer’s instructions. The cRNA concentration was adjusted to 1 µg/µL. Preparation and injection of *X. laevis* oocytes were completed as previously described [21]. The oocytes were injected with 30 nL of a solution containing the cRNA of interest. Injected oocytes were maintained at 19 °C in a survival solution containing (in mM): NaCl, 96; KCl, 2; CaCl_2_, 1.8; MgCl_2_, 1; Hepes, 5; Na-pyruvate, 2.5; and gentamycin, 0.025, pH = 7.2 with NaOH, renewed daily.

### 2.3. Electrophysiology

Expressed currents were recorded at room temperature using the two-electrode voltage-clamp method 1–3 days after injection. Electrodes were pulled from borosilicate glass (Warner, ref GC150T10) and filled with 3 M KCl. Oocytes were clamped at −60 mV, and GABA-gated currents were recorded under voltage-clamp with a GeneClamp 500B amplifier (Molecular Devices, San Jose, CA, USA) and digitized with a Digidata 1200 converter (Molecular Devices) using Clampex software (version 7.0, Molecular Devices). Drugs were applied using a gravity-driven perfusion system at a speed of 2 mL/min. 

**Figure 1:**
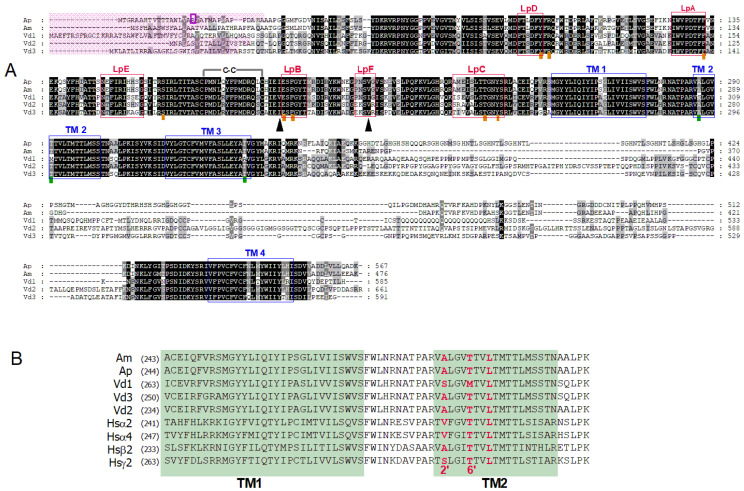
(**A**) Alignment of *Ap*-RDL1 (AXY92182.1), *Am*-RDL (AJE68941.1), *Vd*-RDL1 (AVY53069.1), RDL2 (AYR04938.1) and RDL3 (AVY53071.1) amino acid sequences. Loops involved in GABA binding (LpA-F) are boxed in red, transmembrane segments (TM1-4) are boxed in blue. Amino acids that have been shown to be involved in GABA binding are underlined with a brown square and those in non-competitive antagonists binding with a green square. The location of the C->R substitution in our *Ap*-RDL1 clone is boxed in purple. (**B**) Enlargement of the TM1-TM2 sequence alignment with key amino acids for non-competitive antagonists at 2′, 6′ and 9′ locations bolded in red. Human sequences for α_2_, α_4_, β_2_ and γ_2_ GABA-A receptor subunits are also presented.

For single-channel recordings, the oocyte vitelline membrane was manually removed using forceps after immersion in a hypertonic solution containing NaCl, 200 mM; HEPES (N-(2-Hydroxyethyl)piperazine-N′-(2-ethanesulfonic acid)), 10 mM; pH 7.2 adjusted with NaOH. The oocyte was then placed in the recording chamber filled with a depolarizing OSO-100 solution with KCl, 100 mM; HEPES, 5 mM; EGTA, 10 mM pH 7.2 adjusted with KOH. Firepolished and coated (Sylgard^®^) patch pipettes had a resistance of 8–12 MΩ when filled with the pipette OSOS-100 solution. Cell-attached patch-clamp currents were recorded with an Axopatch 200B amplifier (Molecular Devices), low-pass filtered at 2 kHz and digitized at 10 kHz using a Digidata 1200 interface and stored on a computer using the Clampex software (version 7.0). The liquid junction potential was 1–3 mV and was neglected. Currents were analyzed with the Clampfit software (version 10). Well-resolved channel openings were detected by a threshold analysis set at 50% of the elementary current. Single-channel amplitudes were extracted using Gaussian fits of the amplitude histograms obtained at different voltages (see Figure 1), and channel conductance was calculated from a linear fit of the current–voltage curve constructed with these amplitudes.

### 2.4. Solutions and Drugs

Oocytes were perfused with the following ND solution (in mM): NaCl, 96; KCl, 3; CaCl_2_, 0.5; MgCl_2_, 1; HEPES, 5, with pH set at 7.4 with NaOH at ~1 mL/min. The reversal potential of the *Ap*-RDL receptor was measured in solutions containing (in mM): NaCl, Na-acetate or TEACl (tetraethylammonium chloride), 100; HEPES, 5 with pH set at 7.4, with NaOH or TEAOH. GABA was dissolved daily from a stock solution at 500 mM in water to the final concentration, usually 50–100 µM, except for dose–response curves. Fipronil or picrotoxin were dissolved at the final concentrations from stock solutions in DMSO (dimethylsulfoxide) at 200 and 10 mM, respectively. The final DMSO concentration was always less than 0.2%. 

### 2.5. Analysis

Sequence alignment was completed using Vector NTI tools.

For dose–response curves, peak current amplitudes were first measured as the current at the peak of the response to GABA perfusion minus the holding current without GABA (using Clampfit version 10), and then normalized according to the maximal amplitude, usually recorded at the highest dose. These normalized amplitudes were plotted against log [GABA] concentrations and then were fitted with a logistic function using Origin 6.0 (Microcal Software):

Rel.Cur. = 1/(1 + 10^(log(EC_50_) − log ([GABA])) × p))

With Rel.Cur. the current amplitude at each concentration relative to the current recorded at the highest concentration; EC_50_, the concentration for half-maximum effect, log ([GABA]) the logarithm of the GABA concentration, and p a slope factor.

For fipronil and picrotoxin inhibition curves, the GABA concentration was 100 µM. For dieldrin, the GABA concentration was 100 µM (for *Ap*- and *Am*-RDL, *Vd*-RDL2, -RDL3, and *Hs*-GABA-A-α_1_β_2_γ_2_ and α_4_β_2_γ_2_) or 300 µM (for *Vd*-RDL1 and *Hs*-α_2_β_2_γ_2_) Fipronil and pirotoxin were added to the GABA solution at the desired concentration, and amplitudes were measured as above, except for normalization which was completed relative to the current recorded without an inhibitor. The concentration required to inhibit 50% of the GABA response (IC_50_) and the slope factor p was determined with a logistic function using Origin 6.0:

Rel.Cur. = 1 − 1/(1 + 10^((log(IC_50_) − log ([Inhib])) × p))

With Rel.Cur. the current amplitude at each concentration relative to the current recorded in the absence of antagonist; IC_50_, the concentration for half-maximum inhibition, log ([Inhib]) the logarithm of the inhibitor concentration, and p a slope factor.

For dieldrin, the effect of the compound needs several stimulations to reach the steady-state (around 12–15, and the above protocol cannot be used without introducing uncertainties due to possible desensitization that could take place during this long protocol (7–10 doses with 15 depolarizations every 45 sec for each, would last more than one hour). We, therefore, chose to analyze the kinetics of a single dose and extracted the time constant (Tau Block) and the amount of block (Rel. B) obtained using a mono exponential function in Origin:

Rel.Cur. = Amp × exp(−t/Tau.Bock) + R(1)

With t, the time of the recording; Rel.Cur., the current amplitude at each concentration relative to the current recorded in the absence of antagonist; Amp, the amplitude of the exponential, Tau Block, the time constant of the exponential, and R the residual non-inhibited current at the steady-state of the effects. The relative block (Rel.B) is estimated as 1-R expressed in %.

Data are presented as the means ± S.E.M of *n* individual oocytes. The difference between two means was tested using the non-paired Student’s *t* test (implemented in Origin 6.0) and declared to be statistically significant for *p* values < 0.05. 

## 3. Results

We cloned the RDL1 subunit from the pea aphid *A. pisum*. The amino acid sequence is identical to that reported by del Villar et al., denoted RDL1ad [8], with the exception of a single cysteine at position 18 found to be an arginine in our sequence (Figure 1A, purple box). These first 50 amino acids are in a region that is poorly conserved among species (Figure 1A) where potential signal peptides were predicted in each of these sequences (underlined in pink). This sequence has not been reported to play any particular role for receptor and channel functions so far, and our pharmacological characterization did not evidence any differences between the two RDL1 subunits (from del Villar et al. [8] and this work; see below). 

The amino acid alignment of the five RDL sequences from *A. pisum*, *A. mellifera* and *V. destructor* (Figure 1A) shows an almost complete conservation of the two cysteines involved in the cysteine-loop typical of the pentameric ligand-gated ion channels, the amino acid sequences that form the GABA binding sites (loop A–F, red boxes in Figure 1A) and those that form the transmembrane chloride channel (TM1-4, blue boxes in Figure 1A). In particular, the amino acids expected to be in direct contact with the GABA molecules (underlined by orange rectangles [22,23]) and those involved in chloride selectivity within the channel pore [24,25], in the second transmembrane segment (TM2) with the ‘PAR’ sequence and the amino acids at the 2′ and 6′ positions underlined by green rectangles in Figure 1A,B (relative numbering according to [25,26]) are identical between the *A. pisum* and the *A. mellifera* receptors. However, comparison with the three *Vd*-RDL subunits that can form homomeric receptors [7] highlights several differences in these regions. Some sequences are common to all three *Vd*-RDL subunits (in LpD, LpA, LpB), while others (LpF, TM4) can be found in only two subunits. Of particular interest are the positions 2′ and 6′ in the TM2, two amino acids, alanine and threonine (AT), involved in the binding of open channel blockers [1,4,27]. A serine is found at position 2′ in the *Ap*-RDL2 receptor (not shown, but see [8]) but also in the *Vd*-RDL1 subunit where the most common sequence A2′ and T6′ is replaced by S and M (Figure 1B). Other changes can also be noticed in TM1 and TM3 segments and suggested, as already noted by Menard et al., particularities in channel conductance or sensitivity to non-competitive antagonists [7,25,28].

**Figure 2:**
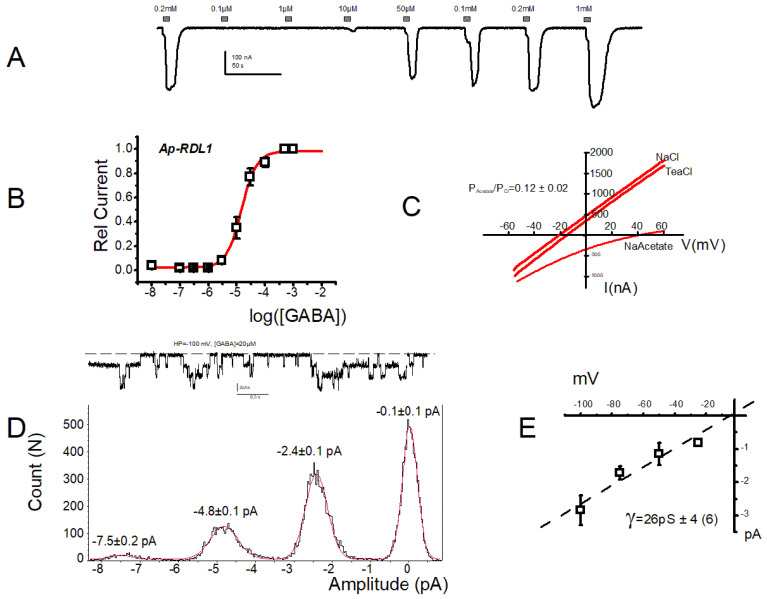
Biophysical characterization of the (*Ap*-RDL1 receptor expressed in *X laevis* oocytes. (**A**) Current trace showing the response of *Ap*-RDL1 to increasing concentrations of GABA from 0.1 µM to 1 mM at a membrane potential of −60 mV. (**B**) Full dose–response curve of *Ap*-RDL1 for GABA. The smooth line represents the best fit to a logistic function. See Table 1 for EC_50_ and *p* values. (**C**) Representative current traces showing the response of the *Ap*-RDL1 to voltage ramps from −60 to +60 mV in NaCl, TEACl or Na acetate solutions. The calculated relative permeability Pacetate/Pchloride was 0.12 ± 0.02, n = 6. (**D-top**) Single-channel traces recorded in cell-attached patches of oocytes expressing the RDL1 GABAr subunit. The pipette solution contained ND and GABA at 100 µM, and the external solution was OSO-100 (see methods). Note the presence of (at least) 3 channels under the patch. (**D**-**bottom**) The amplitude histogram of the trace is shown with the value of the amplitude of each peak deduced from the multi-Gaussian fit (red curve). (**E**) Single-channel current-voltage curve obtained at different voltages from 6 patches as shown in (**D**-**top**) The single-channel conductance is deduced from a linear regression through the experimental points: 26 ± 4 pS.

Injection of *Ap*-RDL1 cRNA into *Xenopus* oocyte gave rise 1–4 days later to robust GABA-induced current responses (Figure 2A) of several hundreds of nanoAmperes (nA). Building the dose–response curve to GABA from these current traces allowed to determine an EC_50_ of 21.2 ± 2.7 µM (n = 20, Figure 2B and Table S1 in supplementary information). This value was close to that reported by del Villar et al. for the *Ap*-RDL1ad (23 ± 2 µM). We then verified the chloride selectivity of the *Ap*-RDL1 receptor using solutions where sodium or chloride ions were substituted by tetraethylammonium (TEA) or acetate, respectively. As seen in Figure 2C, replacing NaCl by TEACl in the perfusing solution (i.e., changing the external cation), did not modify neither the amplitude nor the reversal potential of the GABA-induced currents, with reversal potentials of −12.9 ± 1 mV (n = 5) and −7.4 ± 2.5 mV (n = 6), respectively (*p* > 0.05). However, replacing NaCl in the solution with Na acetate (i.e., changing the external anion) noticeably modified the current shape and the current reversal potential from −12.9 ± 1 mV (n = 5) to 45.7 ± 4.5 (n = 6), respectively (*p* < 0.05). *Ap*-RDL1 is, thus, a true chloride channel with a relative permeability Pacetate/Pchloride of 0.12 ± 0.02 (n = 6), close to that obtained for honeybee *Am*-RDL (0.10 ± 0.02, n = 6, *p*> 0.05), as expected from their identical transmembrane TM2 pore-lining sequences.

We then recorded single RDL channel activity in the presence of 20 µM GABA and in a solution containing 100 mM KCl to nullify the oocyte membrane potential. In cell-attached mode, at a pipette potential of +100 mV and with GABA in the pipette, multiple well-defined openings could be visualized, suggesting the existence of at least 3 channels under the pipette tip (Figure 2D). Fitting the amplitude histogram of this current trace with the equation for 4 Gaussians gave the following pic values of −0.1 ± 0.1, −2.4 ± 0.1, −4.8 ± 0.1 and −7.5 ± 0.2 pA. We undertook the same analysis at different voltages and plotted the single channel current–voltage curve (Figure 2E), using the amplitude differences between the first two peaks, corresponding to the opening of a single- channel. The linear fit of this current–voltage curve gave a single channel conductance of 26 ± 4 pS (n = 6 patches), a value close to the conductance of the *Am*-RDL (29 ± 2 pS, n = 7) and *Dm*-RDL (22 pS) subunits expressed in oocytes [10,24]. These data fit with the fact the *A. pisum* and *A. mel* receptors share 85% of identical amino acid sequences, with the key regions for GABA binding and channel selectivity (loop A-F and TM1-4) being 100% identical [25].

With these five RDL receptors from insect or mite species considered either as pest or as beneficial (*Ap*-RDL1, *Vd*-RDL1 to 3 and *Am*-RDL), we decided to perform a parallel pharmacological characterization, including responses to the agonist GABA, and the antagonists fipronil, picrotoxin and dieldrin, in similar conditions (i.e., after expression in *X. laevis* oocytes) to identify differences that could be of physiological and/or pharmaco-toxicological interest. We added to this study two human GABA-A receptors (α_2_β_2_γ_2_ and α_4_β_2_γ_2_), for comparison with mammalian receptors.

**Figure 3:**
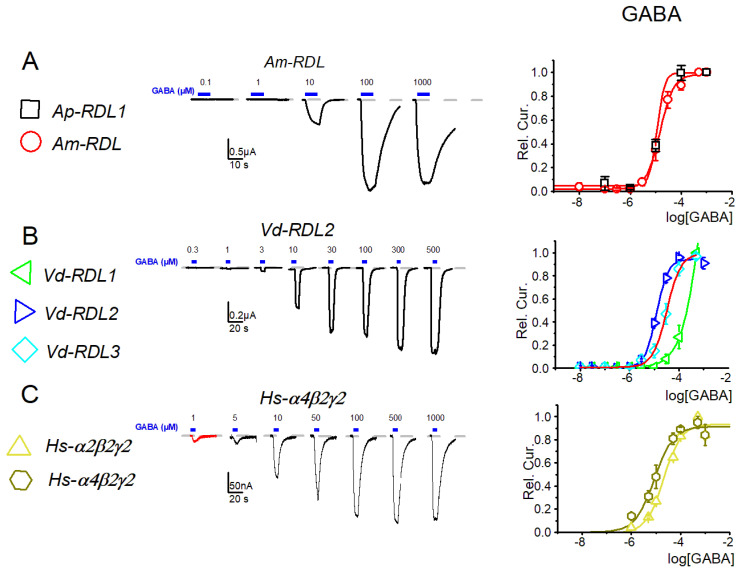
Dose–response curves for GABA of seven GABA-A receptors: RDL of *A. pisum* and *A. mellifera* (**A**) RDL1, RDL2 and RDL3 of *V. destructor* (**B**), and α_2_β_2_γ_2_ and α_4_β_2_γ_2_ of *H. sapiens* (**C**). Left exemplar current traces from 1 type of receptor, right full dose–response curves of the seven GABAr types. The EC_50_ are summarized in Supplementary Table S1.

The sensitivity of *Ap*-RDL to GABA, as determined above, was close to that of *Am*-RDL (EC_50_ of 19.3 ± 3.5 µM, n = 13 see Figure 3) and of *Vd*-RDL2 and *Vd*-RDL3 (14.8 ± 1.8, n = 13 and 43.1 ± 10.4 µM, n = 9, respectively). These values were relevant with the conservation within these receptors of the six loops in the RDL sequence that form the cavity where GABA binding occurs [29], and, in particular, of the seven amino acids directly involved in the binding of the GABA molecule to the receptor (Figure 1A). The *Vd*-RDL1 subunit, however, displayed a completely different EC_50_ for GABA: 269 ± 41 µM (n = 10, Figure 3). 

**Figure 4:**
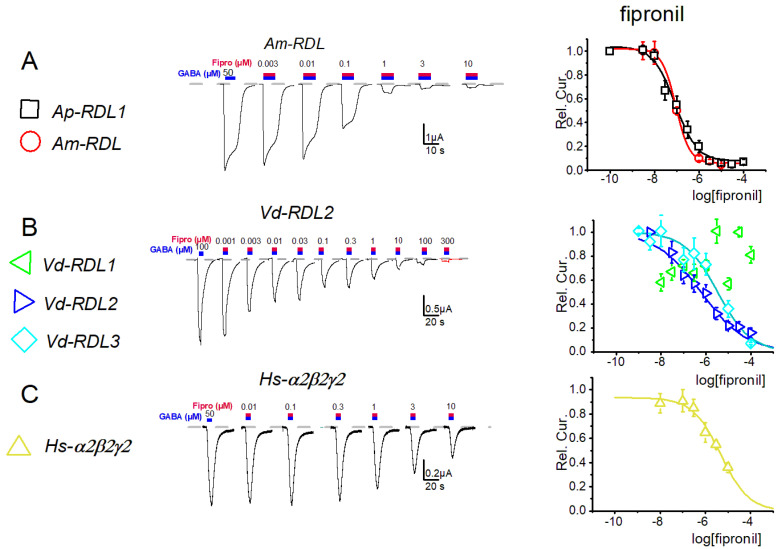
Dose–response curves for fipronil obtained for the seven GABA-A receptors: RDL from *A. Pisum* and *A. mellifera* (**A**) RDL1, RDL2 and RDL3 from *V. destructor* (**B**), and α_2_β_2_γ_2_ and α_4_β_2_γ_2_ GABA-A receptors from *H. sapiens* (**C**). (**Left**), exemplar current traces from 1 type of receptor, (**Right**), full dose–response curves. The IC_50_ are summarized in Supplementary Table S1.

**Figure 5:**
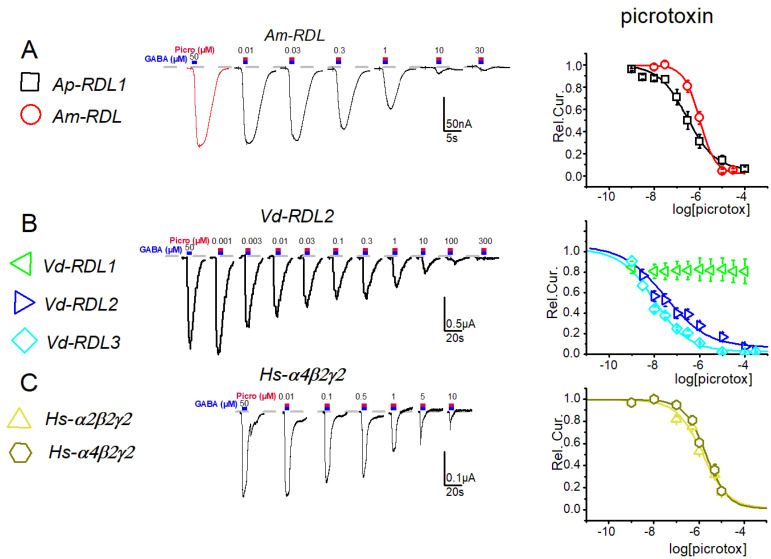
Dose–response curves for picrotoxin obtained for the seven GABA-A receptors RDL from *A. pisum* and *A. mellifera* (**A**), RDL1, RDL2 and RDL3 from *V. destructor* (**B**), and α_2_β_2_γ_2_ and α_4_β_2_γ_2_ from *H. Sapiens* (**C**). (**Left**): representative current traces from 1 type of receptor; (**Right**): full dose–response curves. The IC_50_ are summarized in Supplementary Table S1.

**Figure 6:**
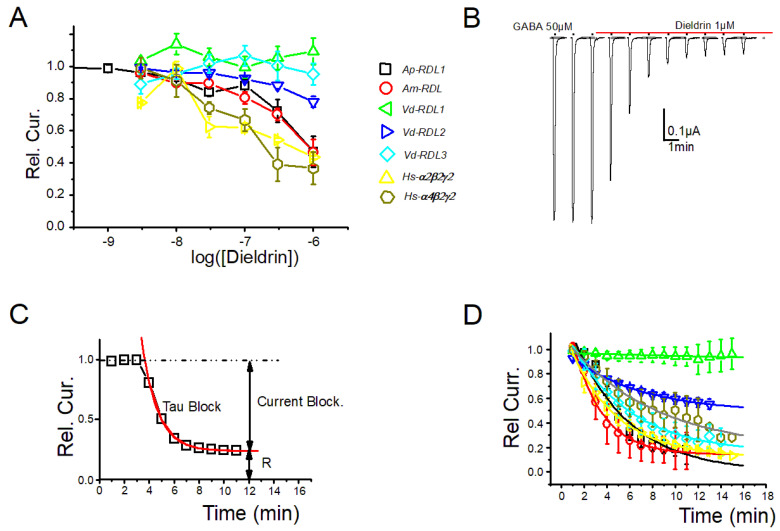
(**A**) Dose–response curves for dieldrin in conditions similar to Figure 4 and Figure 5 (single and simultaneous application of GABA and each dieldrin concentration) obtained for the seven GABA-A receptors: RDL fom *A. pisum* and *A. mellifera*, RDL1, RDL2 and RDL3 from *V. destructor*, and α_2_β_2_γ_2_ and α_4_β_2_γ_2_ from *H. sapiens*. (**B**) Representative current traces of *Ap*-RDL1 in response to repetitive application of GABA (50 µM) before and during continuous perfusion of dieldrin (1 µM). (**C**) The time course of the inhibition by dieldrin of these GABA-induced current amplitudes ((Rel.Cur.) can be approximated by a single exponential that allows to extract Rel.B (relative current block, see Materials and Methods) and a time constant Tau-Block. (**D**) The fit of the kinetics of the response of seven GABA-A receptors: RDL of *A. Pisum* and *A. mellifera*, RDL1, RDL2 and RDL3 of *V. destructor*, and α_2_β_2_γ_2_ and α_4_β_2_γ_2_ of *H. sapiens* to dieldrin gave Tau-Block and Rel.B for each receptor (see Figure 7).

**Figure 7:**
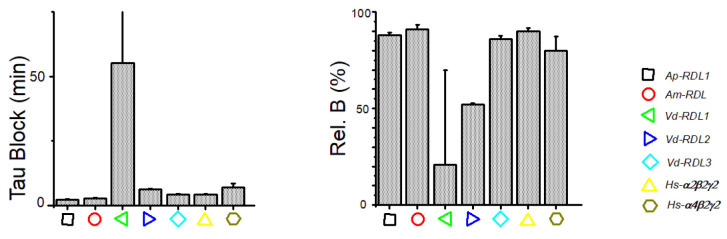
Tau-Block (**Left**) and Rel.B (**Right**) values for the inhibition by dieldrin of each RDL receptor from *A. pisum* and *A. mellifera*, RDL1, RDL2 and RDL3 from *V. destructor*, and α_2_β_2_γ_2_ and α_4_β_2_γ_2_ from *H. sapiens* deduced from the mono-exponential fit of the responses shown in Figure 6D.

A pharmacological analysis was then initiated using either natural, picrotoxin or synthetic, fipronil and dieldrin, insecticides (Figure 4, Figure 5, Figure 6 and Figure 7). Analysis of the sensitivity of these 7 receptors to the phenyl pyrazol fipronil (Figure 4) gave quite close IC_50_ values (range 0.3–3 µM, see Supplementary Table S1) for the *Ap*-RDL1, *Vd*-RDL2, *Vd*-RDL3 and *Hs*-α_2_β_2_γ_2_ GABAr. 

These values were also closed to those previously published (0.18 ± 0.1 and 0.72 ± 0.2 µM for *Ap*-RDL1 and *Ap*-RDL2, [8]; 1.1 ± 0.2 µM for *Hs*-α_1_β_2_γ_2_ [30], for example). The only two exceptions were the values obtained for *Vd*-RDL1 with a very low (almost no block at 30 µM) sensitivity to fipronil (Figure 4) and for *Am*-RDL exhibiting the highest sensitivity to fipronil (0.07 ± 0.2 µM). A poorly fipronil-sensitive *Vd*-RDL1 receptor was also reported in the work of Menard et al. [7] although their IC_50_ was smaller. Part of this insensitivity of *Vd*-RDL1 probably lies on the amino-acids at positions 2′ and 6′ in the second transmembrane region of the channel (TM2, Figure 1A) that have been shown to be important for the binding of open-channel blockers in the pore. Their mutation, either « natural », in resistant insects (as is the case here for *Vd*-RDL1 where amino acids SM replace amino acids AT found in the other RDL subunits at positions 2′ and 6′, respectively), or produced in the lab, affect fipronil inhibition greatly [4,7,31,32]. It should also be noted that the higher sensitivity of *Am*-RDL could account for the high toxicity of this insecticide for honeybees in vivo [33].

The analysis of the sensitivity of these receptors to picrotoxin gave results similar to those obtained with fipronil (see Figure 5 and Supplementary Table S1). The *Ap*-RDL1, *Am*-RDL, *Vd*-RDL2 and *Hs*-α_2_β_2_γ_2_ and *Hs*-α_4_β_2_γ_2_ displayed close EC_50_ (from 0.25 to 1.7 µM) for picrotoxin whereas the *Vd*-RDL1 appeared to be insensitive to this product. A surprisingly low IC_50_ (0.04 µM) was found for the *Vd*-RDL3. It should be noted that del Villar et al. [8] and Menard et al. [7] did not use picrotoxin in their works. This particular sensitivity of *Vd*-RDL3 cannot be explained by the presence of these two amino acids at positions 2′ and 6′ in the TM2, since the *Vd*-RDL3 subunit sequence is perfectly similar to the *A. mellifera* and *A. pisum* sequences from position −6′ to +16′ before and within the TM2 sequence. A N-7′ to H and A20′ to S are the only differences that can be noticed in this region, but similar changes are also present in the *Vd*-RDL1 subunit that displays a very high IC_50_ for picrotoxin (only 20% block with 100 µM, Figure 5B) and are not in a position known to be important for open channel block [31]. The reasons for this high sensitivity are therefore unknown and require further studies. 

The use of dieldrin was a little more challenging since the time course of the inhibition during successive pulses at the same dose (see Figure 6B) was so slow (steady-state was reached in more than 10 min) that the dose–response curves obtained using the first or second stimulation at each dose did not reflect steady-state inhibition (Figure 6A). Realizing dose–responses curves at the steady-state effect (i.e., using the 10th episodes for each dose), however, requires a recording time of more than an hour; thus increasing current run-down/desensitization. We therefore decided to limit our analysis at the extent and the time course of the block at a single dose of 1µM. As seen in Figure 6C, this analysis revealed, nonetheless, marked differences between the different types of RDL or GABA-A subunits tested. This was seen on both the time constants and the steady-state relative block (Tau-Block and Rel.B., respectively, Figure 6D and Figure 7) extracted from these curves after a fit using a mono-exponential decay. 

These values for each receptor are shown in Figure 7. The time constants of all these receptors are grouped around 3 min (range 2–6 min), except for the *Vd*-RDL1 with a clearly slower decline and a Tau-Block that was hard to evaluate and were fitted to around 55 min. The steady-state block was also very low for *Vd*-RDL1r and evaluated close to 20%. *A. pisum*, *A. mellifera*, *V. destructor* RDL3 and *H. sapiens* GABAr were almost completely blocked (range 80–91%). The *V. destructor* RDL2 was a little less affected by dieldrin with an inhibition reaching only 51 ± 1%. As noted above, compared to *A. pisum* and *A. mellifera* receptors, the *V. destructor* RDL1 and RDL2 have modifications in the nature of the residues at positions 20′ and 21′ on the top of the TM2 sequence. However, although these residues line the channel pore [34], they play no role in binding pore-blocking compounds [31]. The reasons for this lower sensitivity should, therefore, be found outside the TM2 and will need in-depth mutational analysis.

## 4. Discussion

**Figure 8:**
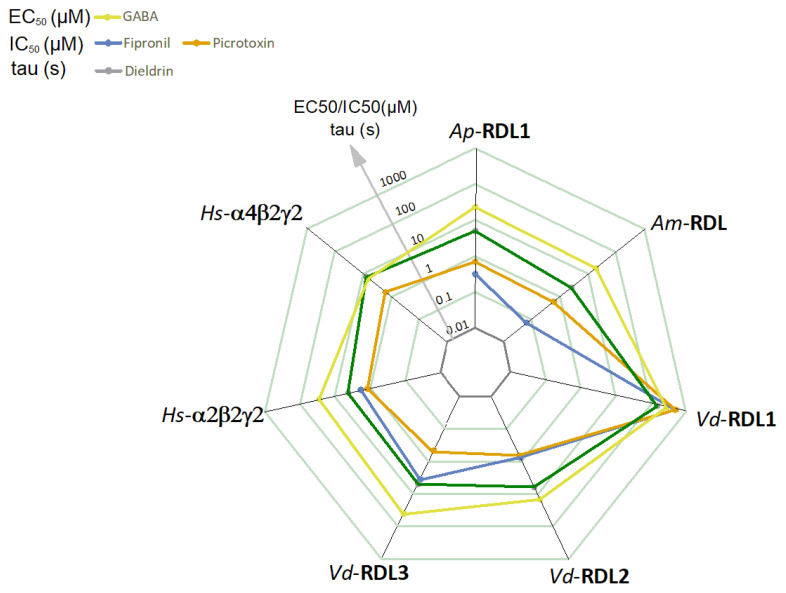
Radar graph summarizing the pharmacological data obtained with the different GABA-A receptors during this study. GABA (EC_50_ in µM and yellow), fipronil (IC_50_ in µM and blue), picrotoxin (IC_50_ in µM and orange) and dieldrin (Tau-block in seconds and in green) are shown; for values see Supplementary Table S1.

All these data were compiled on a radar graph shown in Figure 8, where EC_50_ for GABA, IC_50_ for fipronil and picrotoxin and time constant of block for dieldrin are shown for all seven GABAr. It is worth noting that when published results for a given receptor and a given molecule are available (EC_50_ for GABA and IC_50_ for fipronil, see above), the results are quite similar to ours, despite sometimes slight differences in the experimental protocols.

If we now look at the GABA response (yellow trace), the first thing we can notice is that all these receptors have a relatively similar sensitivity to GABA (EC_50_ in the same log range) except the *V. destructor* RDL1 subunit which is clearly less sensitive as noted above. In the absence of structural information on the *V. destructor* RDL1 subunit, a possible explanation for this insensitivity may only rely on the inspection of the amino-acid sequences of these subunits. While the phenylalanine in loop B and the tyrosine in loop C (F192 and Y240 in the *A. pisum* sequence) of the GABA binding pocket that form cation-π interaction with GABA [22] are conserved in all sequences, in the *Vd*-RDL sequences, some differences exist in loops D, E, F and C. More precisely, in loops C, E and F each *Vd*-RDL subunit has its own arrangement of amino acids that could influence their EC_50_ for GABA. One can notice that among the non-conserved amino acids present in these four loops, isoleucine in loop B and valine in loop F (black arrowhead in Figure 1A) are conserved in all the sequence except in *Vd*-RDL1. In this GABAr, isoleucine and valine are replaced by valine and an isoleucine, respectively. As noted before, the *Vd*-RDL1, as *Vd*-RDL2 and *Vd*-RDL3 sequences, has several other non-conserved residues in these loops, but these two amino acids are unique to *Vd*-RDL1 and although homology modeling and docking do not predict a role in GABA binding [22] they could, nevertheless, constitute a target for future mutagenesis studies, in order to shed some light on this unusual GABA sensitivity. 

Besides any molecular explanation for this behavior, one can easily notice that *Vd*-RDL1 clearly stands out from the other RDL receptors with a very low sensitivity to all the inhibitors tested here. A lower sensitivity to GABA, by preventing receptor opening for a low dose of GABA, should participate, in part, in the insensitivity of the receptor to open channel blockers. The A2′S and T6′M in TM2, the T355A at the end of TM3 or other differences in the loop involved in GABA binding (see Figure 1) may be important, but similar mutations have been described individually in other RDL receptors and their effect has never been shown to be so strong. Moreover, the mutations in the A2′S and S6′M have been shown to play a role in the GABA response, but in the other direction toward an higher sensibility [31]. Therefore, no evident explanation can be drawn from the sole direct inspection of these aligned sequences, but it may help an in-depth mutagenesis approach, to more precisely understand differences in GABA binding and GABAr gating and pharmacology and provide clues on this particular profile. Such information will undoubtedly be important for the search for specific GABAr competitive antagonists which are becoming to be envisaged as potential leads for new classes of insecticides to overcome the increasing resistance to non-competitive antagonists such as fipronil and find more species-specific antagonists [35].

Now, if we excluded the atypical insensitive *Vd*-RDL1, the second thing we can notice is that, after expression in *X. laevis* oocytes, i.e., in similar conditions, the pharmacological profiles of all these receptors from 4 different species are very close, except for the *Vd*-RDL3 and *Am*-RDL receptors that show distinctive sensitivity to the banned insecticides picrotoxin and fipronil, respectively. These behaviors do not rely on particular residues located within the pore, since the *A. mellifera* TM1-4 amino acid sequences, for example, are identical to those of *A. pisum*. These parallel characterizations, thus, demonstrate that multiple molecular interactions involving residues outside the TM2 participate in the pore structure and finely tune the pharmacological profiles of these receptors. A third interesting point is that this type of representation (Figure 8) clearly underlines interesting specific features between species and molecules. For example, the relative inefficacy of all these products on *Vd*-RDL1 receptors or the very high sensitivity of the *Vd*-RDL3 subunits to picrotoxin. This approach can clearly be helpful in a very early step when one wants to choose a molecule able to protect plants against a given pest or preserve a beneficial species. 

Possible different subunit compositions of RDL with the three other potential GABAr subunits (GRD, LCCH3, GC8916), or even just these subunits, since GRD + LCCH3 have be shown to produce cation-selective receptors opened by GABA [9,36], should, however, also be considered to fully evaluate the in vivo pharmacology of these receptors. The co-expression of RDL and LCCH3 subunits has been demonstrated, for example, in the antennal lobe neurons of *D. melanogaster* by single-cell PCR, suggesting that heteromeric receptors may play a role in regulating synaptic transmission [37]. Post-translational processes, such as alternative splicing and RNA editing [27,38,39], also provide some diversity in the biophysical and pharmacological properties of the GABAr [6] which do result in heterogeneity in the cellular GABAr populations. Finally, the mode of absorption (topic or ingestion), the bio-availability, and the pharmacokinetic parameters of each compound, which are species-specific parameters, must be taken into account together with the pharmacological properties of the related products, the *A. mellifera* having a deficit in detoxification enzymes, for example [40,41].

It should be noted that despite the low sequence conservation (28–33% for α_2_ and α_4_, see Table 1) between the two *H. sapiens* and the insect GABA-A receptors, their pharmacological profiles are similar. The EC_50_ for GABA were 27.4 ± 4.4 and 6.9 ± 2.2 µM for α_2_β_2_γ_2_ and α_4_β_2_γ_2_, respectively. For fipronil, the IC_50_ were 1.8 ± 0.4 µM for α_2_β_2_γ_2_ and for picrotoxin 1.2 ± 0.1 and 1.6 ± 0.2 µM for α_2_β_2_γ_2_ and α_4_β_2_γ_2_, respectively. These values were close to those already found for mammalian α_2_β_2_γ_2_ and α_4_β_2_γ_2_ GABA-A receptors [30]; but also close to the insect/mite values (Figure 8, Supplementary Table S1). They might be due to the fact that these compounds (GABA and fipronil/picrotoxin/dieldrin) target sites that are closely related to their function (GABA recognition and a channel pore with Cl selectivity) and are, thus, quite conserved.

In conclusion, this work provides a comparative characterization of the *A. pisum* GABAr versus those of pests, beneficial insects, or humans. These three types of organisms may all encounter phyto-pharmaceutical products at different levels, but while the presence of the so-call pests can be problematic in some cases, beneficial insects and humans must be preserved. Such studies are, therefore, needed to guide our biosafety choices. These expression studies are focused on the receptor sensitivity only, i.e., the molecular mode of action of insecticides. They, nevertheless, highlight [1] the poor sensitivity to agonists and non-competitive antagonists of the *Vd*-RDL1 receptor and [2] the similarity of pharmacological profiles of the other GABAr, with some exceptions limited to one species toward one product (i.e., *A. mellifera* and fipronil), but do not deal with actual agricultural practices or with pharmacokinetic mechanisms that are also important parameters for accounting for in vivo toxicity. However, in the current context of pesticide reduction policies, they may be useful in the banning process of the most dangerous marketed products and the precocious rejection of new products by providing some clues for in vitro drug selection/rejection. The presence of other subunits in neurons or other cells or possibly different post-translational processes (glycosylation, phosphorylation, RNA-editing) that may affect channel permeation and/or pharmacology may also be considered and included in further studies.

## Figures and Tables

**Table 1:** Percentage of identity between the different RDL subunits.

	*Am*-RDL	*Ap*-RDL1	*Ap*-RDL2	*Hs*-GABA-Aα2	*Hs*-GABA-Aα4	*Vd*-RDL1	*Vd*-RDL2	*Vd*-RDL3
*Am*-RDL		85	86	35	33	59	62	58
*Ap*-RDL1			88	32	29	51	53	52
*Ap*-RDL2				33	29	52	52	52
*Hs*-GABA-α2					58	32	33	33
*Hs*-GABA-α4						28	28	26
*Vd*-RDL1							61	58
*Vd*-RDL2								64
*Vd*-RDL3								

## Data Availability

Please contact the corresponding author.

## Supplementary Materials

The following supporting information can be downloaded at: https://www.mdpi.com/article/10.3390/membranes12050440/s1, Table S1: Pharmacological parameters of insect and human GABA receptors.
