# Supplementary material for: Xenopus Oocytes: A Tool to Decipher Molecular Specificity of Insecticides towards Mammalian and Insect GABA—A Receptors
Source: Membranes (Basel). 2022 Apr 19;12(5):440. doi: 10.3390/membranes12050440 (PMC9146934; doi:10.3390/membranes12050440)
Supplement: Supplementary file 1 [file membranes-12-00440-s001.zip › membranes-1641387-supplementary.pdf]

**Supplementary Table S1**. Pharmacological parameters of insect and human GABA receptors.

| Protein (Accnb) | GABA | | fipronil | picrotoxin |
|---|---|---|---|---|
| | $EC_{50}$ µM) | p | $IC_{50}$ (µM) | $IC_{50}$ (µM) |
| *A. pisum RDL1* | 23.8 ± 2.5(20) | 2.4 ± 0.3 (20) | 0.31 ± 0.11(19) | 0.7 ± 0.2 (12) |
| *A. pisum RDL1ad* AXY92182.1* | 23 ± 1.7 | 1.6 ± 0.1 | 0.64 ± 0.15 | *ND* |
| *A. pisum RDL1bd** | 51 ± 9.1 | 2.0 ± 0.3 | 0.18 ± 0.06 | *ND* |
| *A. pisum RDL2b* AXY92183.1* | 28 ± 5.4 | 1.0 ± 0.1 | 0.72 ± 0.19 | *ND* |
| *A. mellifera RDL AJE68941.1* | 19.3 ± 3.5(13) | 2.1 ± 0.2 (13) | 0.07 ± 0.2(9) | 0.6 ± 0.1(15) |
| *V. destructor RDL1# AVY53069.1* | 269 ± 41 (10) | 2.3 ± 0.2 (10) | *>500µM* | *>500µM* |
| *V. destructor RDL2# AYR04938.1* | 14.8 ± 1.4 (13) | 1.8 ± 0.2 (13) | 0.77 ± 0.25(11) | 0.7 ± 0.5 (14) |
| *V. destructor RDL3# AVY53071.1* | 43.1 ± 10.4 (9) | 2.3 ± 0.1 (9) | 3.64 ± 1.74(5) | 0.6 ± 0.1 (10) |
| *H. sapiens* α2β2γ2[&] | 27.4 ± 4.4(10) | 1.4 ± 0.1 (10) | 1.88 ± 0.39(9) | 1.2 ± 0.1 (4) |
| *H. sapiens* α4β2γ2[&] | 6.9 ± 2.1(21) | 1.4 ± 0.2 (21) | *ND* | 1.6 ± 0.2 (4) |

* : see (8) for results ; #and &see (7) and (14) for partial characterization.
